# Family Socioeconomic Status and Adolescent Depressive Symptoms in a Chinese Low– and Middle– Income Sample: The Indirect Effects of Maternal Care and Adolescent Sense of Coherence

**DOI:** 10.3389/fpsyg.2019.00819

**Published:** 2019-04-11

**Authors:** Fuzhen Xu, Wei Cui, Tingting Xing, Monika Parkinson

**Affiliations:** ^1^School of Psychology, Shandong Normal University, Jinan, China; ^2^School of Psychology and Clinical Language Sciences, University of Reading, Reading, United Kingdom

**Keywords:** adolescent, depressive symptoms, socioeconomic status, maternal care/control, sense of coherence

## Abstract

The current study investigated whether socioeconomic status (SES) was associated with adolescent depressive symptoms through maternal parenting and adolescent sense of coherence (SOC). Using a sample of 1220 Chinese adolescents, it was found that SES, maternal care, and adolescent SOC were positively related to each other and negatively related to adolescent depressive symptoms, respectively. Maternal control was positively related to adolescent depressive symptoms and negatively related to their SOC, but not significantly to SES. By analysis of structural equation modeling, we found that SES was associated with adolescent depressive symptoms indirectly through maternal care separately, as well as through maternal care and adolescent SOC sequentially. This study extended our understanding by showing possible indirect pathways by which family contextual factors and individual internal resources for adolescent depressive symptoms may operate separately and sequentially. The overall results highlighted the need to study adolescent depressive symptoms to find external and internal positive factors for maintaining adolescent emotional health, especially in families with relatively lower income.

## Introduction

Depression is one of the most common internalizing problems in adolescence (Kessler et al., 2001; Pennant et al., 2015; Xu et al., 2015). A survey performed by the National Children’s Bureau (National Children’s Bureau, NCB) in the United States in 2016 showed that the prevalence of adolescent depression was around 11% during 1 year. Wei (2008) also found that approximately 33% of Chinese adolescents experienced depressive symptoms during the past 3 years. Moreover, depression is associated with negative consequences, including academic difficulties, interpersonal dysfunction, as well as health problems (Berndt et al., 2000; Zlotnick et al., 2000; Korczak and Goldstein, 2009), and it may persist into adulthood if left untreated (Aalto-Setälä et al., 2002). Many prior studies have demonstrated that family context and internal resources are potential factors associated with adolescent depression (Elovainio et al., 2012; Moksnes et al., 2012; Sichko et al., 2015).

Within the family context socioeconomic status (SES) and parenting are generally viewed as two fundamental factors. SES is a multidimensional concept and most contemporary researchers agree that it is represented by a combination of family income, parental education and occupational status (Bradley and Corwyn, 2002; Conger and Donnellan, 2007; Ye and Wu, 2012). The link between SES and adolescent depression has been found across both Western and Chinese populations (Amone-P’Olak et al., 2009; Ye and Wu, 2012). According to the family investment model, compared to parents from high–SES, parents with low–SES have less financial capital, lower education and occupational status, making it less likely that they are able to provide good material conditions and engage in positive parenting behaviors, thereby increasing the risk for the development of emotional problems in their adolescent children (Conger and Donnellan, 2007).

Parenting as a proximal family context consists of different dimensions and more specific factors derived from those dimensions. For example, Parker et al. (1979) developed a questionnaire with two dimensions of care (e.g., emotional warmth, closeness and empathy) and overprotection (e.g., control, excessive contact, prevention of independent behavior) to measure parenting practice. Maccoby and Martin proposed a two–dimensional model with responsiveness (e.g., warmth and involvement) and demandingness (e.g., control and monitoring) (see also Piko and Balazs, 2012). And Rapee (1997) regarded parental control and rejection as two main parenting dimensions. A large body of reviews and empirical studies investigating the association of parenting and adolescent depression showed that warmth, care, acceptance and other positive parenting behaviors were negatively linked to depression (Milevsky et al., 2007; Brand et al., 2009a,b; Yap et al., 2014; Wang Y.C. et al., 2015; Little et al., 2017), while negative parenting characterized by harsh, control and neglect were risks for depression (Aunola et al., 2013; Reising et al., 2013; Frazer and Fite, 2016; Murdock et al., 2018). Regardless of the differences in dimension of parenting practice, there is consistency that parental care and control have a close association with individual depression. For instance, parental care has been shown to have a negative association with child depression (Morris and Oosterhoff, 2016; Ono et al., 2017), and Campos et al. (2010) further found that individuals with depression perceived less maternal care. Furthermore, parental control including psychological and behavioral control is a risk factor for depression (Parker, 1983; Frazer and Fite, 2016). Care and control are regarded as two typical dimensions of parenting and it is of particular significance to study their specific effect on Chinese adolescent depression (Wang and Zhang, 2007; Xia and Liang, 2016). In China though a universal two–child policy was carried out recently, the enforcement of the one–child policy since 1979 may result in parenting behaviors that are unique and focus solely on the only child, with both parents trying their best to give their children care, love and concern (Deutsch, 2006). And with the far–reaching influence of collectivism, Confucianism and fixed family hierarchy on Chinese society, children are expected to be obedient to authority and they usually experience more control from parents (Dwairy and Achoui, 2010). Although children who grow up in Chinese cultural setting generally consider parental control as normal, a recent study found that these actions were associated with child problem behaviors (Pomerantz and Wang, 2009).

The family stress model (Conger et al., 2002) and many empirical studies have revealed that SES may affect parenting behaviors. For example, high–income and well educated parents tend to display more care, warmth and supportive parenting (Zhang, 1999; Waylen and Stewartbrown, 2010), while parents with financial stress are more likely to experience depression, which in turn exacerbates problems in parenting (Ponnet et al., 2016; Devenish et al., 2017). In addition, maternal current unemployment has been shown to be associated with depression in adolescence through ineffective child–rearing behavior (Mcloyd et al., 1994). Therefore, it is possible that parental income, education or occupational status may be linked with adolescent depression through parenting behaviors.

According to Benson (2002), internal resources refer to personal characteristics which influence individual development. Recently, sense of coherence (SOC), introduced by Antonovsky (1979) as a personality factor, has attracted more attention, and is defined as the extent to which one has a pervasive, enduring and dynamic feeling of confidence. Antonovsky (1987) suggested that family context was one of the most important factors to have a close association with individual SOC. Children living in high–SES backgrounds usually possess adequate resources (Conger and Donnellan, 2007), and they are more likely to develop stronger SOC compared with children in low SES (Sagy and Antonovsky, 2000). SOC enables people to cope with stress in a health–promoting manner and individuals with higher SOC experience fewer psychological problems (Simonsson et al., 2008). Previous studies have demonstrated the association between SOC and individual depression (Konttinen et al., 2008). For example, Moksnes et al. (2012) analyzed data from 1209 adolescents in Mid–Norway and found that adolescents with a strong SOC exhibited lower levels of depression. In addition, some theoretical and empirical studies found that parenting as another vital family context was related to individual SOC. For example, parental emotional closeness and affection was associated with higher levels of adolescent SOC (García-Moya et al., 2013), and maternal overcontrol was associated with lower levels of adolescent SOC (Wang et al., 2018). Therefore, it is reasonable to expect that SES and maternal care/control parenting would be associated with adolescent depression through their SOC.

Although many valuable findings regarding the relationships between SES, parenting, adolescent SOC and their depression have been reported, potential mechanisms among these relationships need to be further studied. Firstly, the aforementioned studies only examined single indicators of SES rather than a combination of SES with family income, parental education and occupational status. Secondly, the main findings were primarily based on Western cultures. Furthermore, according to Bronfenbrenner’s ecological systems theory (Bronfenbrenner and Morris, 2006), SES and parenting are usually regarded as distal and proximal factors respectively, with the distal factor affecting individual development through the proximal one in the family context. However, it remains unknown whether SES is associated with adolescent SOC and depression through maternal care/control. Additionally, to our knowledge, few studies have examined the relationships between parenting and adolescent SOC, and less is known about the relationships between maternal care/control and adolescent SOC, especially in Chinese society. Finally, given that adolescents in lower–income families are more likely to experience depression compared to adolescents in families with higher income, it is necessary to ascertain the relationships among SES, maternal care/control, adolescent SOC and depression to provide empirical support for improving the mental health of adolescent in low– and middle– income families.

Therefore, the present study examined the direct association between SES and adolescent depressive symptoms and the indirect association through maternal care/control and adolescent SOC in a community sample of Chinese adolescents. We hypothesized that the main variables of SES, maternal care/control, adolescent SOC and depressive symptoms were related to each other. We also hypothesized that SES was associated with adolescent depressive symptoms indirectly through maternal care/control and adolescent SOC separately and sequentially.

## Materials and Methods

### Participants

Participants consisted of 783 middle school students (416 boys and 367 girls) from three public middle schools and 437 high schools students (217 boys and 220 girls) from two public high schools in Jinan, an eastern Chinese city. The mean ages of middle school and high school students were 13.33 years (*SD* = 1.00) and 16.36 years (*SD* = 1.04), respectively. Included participants were living in a two–parent family and the large majority of them came from low– and middle– income families. Nearly 11% of the households had monthly income less than 1000 CNY (approximately $150), 48% between 1000 and 3000 CNY, and 41% more than 3000 CNY (approximately $450). In the sample, 6% of the fathers and 10% of the mothers had completed primary school education or less, 31% of the fathers and 32% of the mothers had a secondary school education, 28% of the fathers and 26% of the mothers had a high school education, 29% of the fathers and 28% of the mothers had a college/university education, and 6% of the fathers and 4% of the mothers had a postgraduate education. In terms of employment, 15% of the fathers and 28% of the mothers were unemployed, 47% of the fathers and 40% of the mothers were working class, and 38% of the fathers and 32% of the mothers had a professional or semi–professional position.

### Procedure

Prior to data collection, we introduced the study aims and procedure to class master teachers. With permission of master teachers, invitation letters including study information and consent forms were delivered to students and their parents. After obtaining written informed consent by master teachers from the participating adolescents and their parents, participants were asked to complete the self–report Chinese–language questionnaires, including SES items, Parental Bonding Instrument (PBI), the Sense of Coherence Scale (SOC–13), and the Center for Epidemiologic Studies Depression Scale (CES–D), during the normal school day. To ensure standardization of procedures across classrooms, members of the trained research team supervised questionnaire administration. The study procedures were conducted following approval by the Institutional Review Board of Shandong Normal University.

### Measures

#### Depressive Symptoms

Adolescent depressive symptoms were assessed using the 20–item Chinese version of the Center for Epidemiologic Studies Depression Scale (CES–D; Chen et al., 2009), which was designed to measure depressive symptomatology in the general population. It consists of four components of depressive symptomatology: somatic symptoms (e.g., “I did not feel like eating; my appetite was poor”), depressed affect (e.g., “I was bothered by things that usually don’t bother me”), positive affect (e.g., “I felt that I was just as good as other people”) and interpersonal relations (e.g., “People were unfriendly”). The CES–D has been widely used and has demonstrated good internal reliability and validity (Hu et al., 2014). Adolescents were required to rate each item on a 4–point scale ranging from 0 (*rarely or none of the time*) to 3 (*most or all of the time*), with higher scores reflecting higher levels of depressive symptoms. In this study, the Cronbach’s alpha coefficient for depressive symptoms was 0.87.

#### Socioeconomic Status

Five indicators were employed to determine SES (Bradley and Corwyn, 2002): monthly family income (henceforth “family income”), parental education, and occupational status. Family income was measured on a 3–point scale ranging from 1 (*1000 CNY or less; approximately $150*) to 3 (*3000 CNY or above; approximately $450*) (Wang M.F. et al., 2015). Parental education was coded on 6–point scale ranging from 1 (*primary school education or less*) to 6 (*postgraduate education*) (Xu et al., 2009). Following recommendations by Fuligni and Zhang (2004), parental occupational status was coded on a 3–point scale ranging from 1 (*unemployed*) to 3 (*professional or semi*–*professional*). Building on previous research (Bradley and Corwyn, 2002), family income, parental education and occupational status were standardized using *z*–scores and then summed so that higher scores reflected higher family SES.

#### Maternal Care and Control

Maternal care and control were assessed using the 23–item Chinese version of the PBI; Yang et al., 2009), which includes care (e.g., “Spoke to me in a warm and friendly voice”), control (e.g., “Did not want me to grow up”) and encouragement of autonomy subscales (e.g., “Let me do those things I liked doing”), separately for mothers and fathers. Maternal care subscale (11 items) and control subscale (6 items) were the primary focus in the current study. The PBI has been frequently used to measure fundamental parenting behaviors and has demonstrated good internal reliability and validity (Chen et al., 2011; Tsaousis et al., 2012). Adolescents reported retrospectively about the parenting received from their mothers using a 4–point response scale ranging from 0 (*very unlike*) to 3 (*very like*). Six items of maternal care subscale were reverse scored. The maternal care and control variables were calculated separately by summing the scores of subscale items, with higher scores reflecting higher levels of maternal care and control. In this study, the Cronbach’s alpha coefficients for maternal care and control were 0.71 and 0.64, respectively. Although the internal consistency of maternal control scale was not high, it was consistent with other studies using the control scale (Chambers et al., 2000).

#### Sense of Coherence

Sense of coherence was assessed via the 13–item Chinese short version of the 29–item Orientation to Life Questionnaire (OLQ; Bao and Liu, 2005), which consists of three subscales: comprehensibility (5 items; e.g., “Do you have the feeling that you are in an unfamiliar situation and don’t know what to do?”), manageability (4 items; e.g., “How often do you have feelings that you’re not sure you can keep under control?”) and meaningfulness (4 items; e.g., “How often do you have the feeling that there is little meaning in the things you do in your daily life?”). The short version of the OLQ has been shown to be valid and reliable (Liu et al., 2006). Adolescents responded on a 7–point scale ranging from 1 (*never happened*) to 7 (*always happened*). Five items were reverse scored. High scores indicated a high level of SOC. In this study, the Cronbach’s alpha coefficient for SOC was 0.85.

### Data Analyses

SPSS 19.0 and MPLUS 7.0 were employed to conduct all analyses. Missing data in MPLUS were accounted for through full information maximum likelihood. Firstly, univariate analysis of variance (ANOVA) was used to examine gender and school stage (middle school and high school) differences in depressive symptoms, maternal care /control and SOC. Secondly, bivariate correlations were conducted to examine associations between main variables. Next, the structural equation model was used to test whether SES was directly associated with adolescent depressive symptoms and indirectly through maternal care/control and/or adolescent SOC separately or sequentially. According to recommendations by Rogers and Schmitt (2004), items from maternal parenting and adolescent depressive symptoms were randomly assigned to three parcels separately and provided three indicators of each latent variable. SES was identified by five indicators: family income, paternal and maternal occupational status, paternal and maternal education. The latent variable of adolescent SOC was created using three indicators including comprehensibility, manageability and meaningfulness. The model was estimated using the following fit indices: the Comparative Fit Index (CFI; >0.90, acceptable; >0.95, good); the Root Mean Square Error of Approximation (RMSEA; <0.08, acceptable; <0.05, good); and the Root Mean Square Residual (SRMR; <0.08, acceptable; <0.05, good) (Hu and Bentler, 1999).

## Results

### Preliminary Analyses

Table 1 contains the means, standard deviations and correlations among all variables. Main variables had skewness and kurtosis that fell within the acceptable range of no greater than ± 2.0 and ± 7.0, respectively (Finney and DiStefano, 2013). Univariate analysis of variance showed that girls scored higher on depressive symptoms, *F*(1, 1161) = 18.91, *p* < 0.001, partial *η*^2^ = 0.02, and maternal control, *F*(1, 1210) = 6.17, *p* < 0.05, partial *η*^2^ = 0.01, and lower on SOC than boys, *F*(1, 1181) = 7.10, *p* < 0.01, partial *η*^2^ = 0.01. Middle school students scored higher on maternal care, *F*(1, 1206) = 11.31, *p* = 0.001, partial *η*^2^ = 0.01, and SOC, *F*(1, 1181) = 28.65, *p* < 0.001, partial *η*^2^ = 0.02, and lower on depressive symptoms than high school students, *F*(1, 1161) = 27.94, *p* < 0.001, partial *η*^2^ = 0.02. SES, maternal care, and SOC were significantly and positively correlated with each other, and they showed significant negative correlations with depressive symptoms; maternal control was significantly and positively correlated with depressive symptoms and negatively with SOC, but there was no significant correlation with SES. Thus, maternal control was excluded in later analyses.

**Table 1:** Descriptive statistics and correlations on study variables.

	Girls	Boys	*F*	MS	HS	*F*	1	2	3	4	5
				
	*M* (*SD*)	*M* (*SD*)		*M* (*SD*)	*M* (*SD*)						
1. Depressive symptoms	16.74 (9.36)	14.24 (8.64)	18.91^∗∗∗^	14.38 (8.92)	17.32 (9.07)	27.94^∗∗^	-					
2. Maternal care	25.15 (5.66)	24.96 (5.53)	0.26	25.45 (5.70)	24.33 (5.33)	11.31^∗∗^	-0.44^∗∗^	-				
3. Maternal control	4.94 (3.40)	4.50 (3.20)	6.17^∗^	4.63 (3.26)	4.84 (3.37)	0.96	0.29^∗∗^	-0.31^∗∗^	-			
4. SOC	58.11 (12.47)	60.22 (13.18)	7.10^∗∗^	60.71 (13.37)	56.54 (11.51)	28.65^∗∗^	-0.69^∗∗^	0.48^∗∗^	-0.32^∗∗^	-		
5. SES	-	-	-	-	-	-	-0.15^∗∗^	0.19^∗∗^	-0.02	0.13^∗∗^	-	


*SES, socioeconomic status; SOC, sense of coherence; MS, middle school; HS, high school; ^∗^*p* < 0.05; ^∗∗^*p* < 0.01; ^∗∗∗^*p* < 0.001.*

### Structural Equation Model Analyses

**FIGURE 1:**
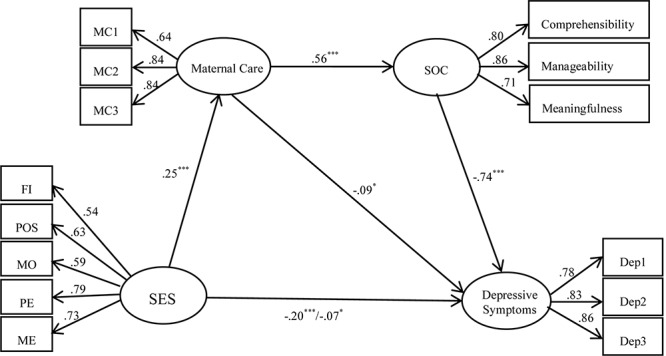
The indirect effects of maternal care and SOC in the relationships between SES and adolescent depressive symptoms. FI, family income; POS, paternal occupational status; MOS, maternal occupational status; PE, paternal education; ME, maternal education; SES, socioeconomic status; SOC, sense of coherence; –0.20^∗∗^, total effect; –0.07^∗^, direct effect; ^∗∗^*p* < 0.01; ^∗∗∗^*p* < 0.001.

First, the measurement model including four latent variables and fourteen observed variables was tested and it provided a good fit to the data, *χ*^2^(71) *=* 415.16, CFI = 0.957, SRMR = 0.033, and RMSEA = 0.063. We found factor loadings were significant for indicators on latent variables, and all latent variables from the measurement model were significantly related with each other. Next, a structural model was used to examine the indirect effects of maternal care and adolescent SOC on the relationship between SES and adolescent depressive symptoms. Figure 1 shows the final model and standardized regression coefficients. Although the original model fitted well, the direct effect of SES on adolescent SOC was not significant, β = 0.01, *SE* = 0.03, *p* > 0.05, and SES was not associated with adolescent depressive symptoms through adolescent SOC, β = –0.01, 95% CI = [–0.064, 0.004], *SE* = 0.03, *p* > 0.05. Therefore the path from SES to adolescent SOC was removed to make the model parsimonious and the new model was examined. The new model also fitted well, *χ*^2^ (68) = 205.99, CFI = 0.983, SRMR = 0.028, and RMSEA = 0.041. The total effect of SES on adolescent depressive symptoms was significant, β = –0.20, *SE* = 0.03, *p* < 0.001. After accounting for maternal care and adolescent SOC, the direct effect of SES on adolescent depressive symptoms was also significant, β = –0.07, *SE* = 0.03, *p* < 0.05. The specific indirect effect of SES on adolescent depressive symptoms through maternal care was significant, β = –0.02, 95% CI = [–0.043, –0.004], *SE* = 0.01, *p* < 0.05, effect size = 10%, as was the specific indirect effect of SES on adolescent depressive symptoms through maternal care and adolescent SOC sequentially, β = –0.11, 95% CI = [–0.137, –0.075], *SE* = 0.02, *p* < 0.001, effect sizes = 55%. The combination of SES, maternal care and adolescent SOC accounted for 65% of the variance in depressive symptoms (*R*^2^ = 0.65, *p* < 0.001).

Given that there were gender and school stage differences in the outcome variables, we tested whether indirect pathways were different for girls versus boys and for younger versus older adolescents using multiple–group analysis. We first examined measurement invariance across groups by comparing the fit of a constrained measurement model (where factor loadings were fixed across groups) with the fit of an unconstrained model (where factor loadings were allowed to vary across groups). Then we tested the structural equivalence by comparing a constrained structural model (where all pathways were constrained to be equal across groups) with the constrained measurement model. According to Cheung and Rensvold (2002), if the difference of CFI (ΔCFI) is less than 0.01, this suggests model invariance. Results found that for gender, measurement invariance of ΔCFI = 0.00, and structural equivalence of ΔCFI = 0.001 were obtained. For the school stage, both the measurement invariance, ΔCFI = 0.00, and structural equivalence, ΔCFI = 0.002, were verified as well. Therefore, the indirect pathways did not differ for boys and girls and for younger and older adolescents. That is to say, gender and school stage could not moderate the indirect relationship.

## Discussion

The present study extended our understanding of the underlying links between family SES and adolescent depressive symptoms in Chinese culture by examining whether family SES was associated with adolescent depressive symptoms indirectly through maternal parenting and adolescent SOC using a Chinese low– and middle– income sample. The results partially supported our hypotheses, suggesting that SES was associated with adolescent depressive symptoms not only through maternal care separately but also through maternal care and adolescent SOC sequentially.

Consistent with the previous findings (Vandervalk et al., 2004; Wang Y.C. et al., 2015), in this study girls reported more depressive symptoms than boys and students in high school reported more depressive symptoms than those in middle school. The current study also found that girls reported more maternal control and lower SOC than boys and middle school students reported higher SOC and more maternal care than those in high school. As this is a Chinese sample, it was possible that Chinese mothers were influenced by traditional Chinese notions of gender roles expectation, which stated that boys should be encouraged to be independent whereas girls should be granted less autonomy and more control (Shek, 2006). The extant research (Moksnes et al., 2012, 2013) showed consistent findings for gender difference on SOC, suggesting that relative to boys, girls tended to view stress they encountered in the environment as less controllable and organized their own resources ineffectively. In the present study, lower SOC, less maternal care and more maternal control were associated with higher levels of depressive symptoms, which was useful to explain why girls reported more depressive symptoms than boys and why students in high school reported higher level of depressive symptoms than those in middle school. One study with a sample of 40– to 70–year–old adults found that SOC tended to increase with age (Eriksson et al., 2007), but it is difficult to draw any comparisons from the current findings given that the population in the two studies varies significantly. Thus, further work to explore the developmental tendency of SOC would be warranted.

The results of this study suggested that the lower the family SES, the less maternal care was displayed to their children and the higher the level of adolescent reported depressive symptoms. Similar results can be found in previous studies, which show that parents with low–SES experience more depression and anxiety themselves (Phongsavan et al., 2006) and are more like to behave toward their children in punishing and less caring ways, and these children are also more likely to experience internalizing problems (Bøe et al., 2014). Although our findings were similar to previous studies, the results need to be interpreted with caution given the weak indirect effect of maternal care on the relationship between SES and adolescent depressive symptoms.

Contrary to our expectations and previous studies conducted in other cultures (Hoff et al., 2002), this study did not find direct association between SES and maternal control, which may be partly due to particular Chinese culture relevant to child–rearing. To some extent, maternal control is similar with *guan*, which is the traditional Chinese parenting notion of “more love and more discipline” (Chao, 1994). Chinese mothers, regardless of their family SES, are generally inclined to exert *guan* in their children’s lives. Additionally, the lack of association also suggested that there may have been other variables our study did not include (e.g., maternal emotional problems) through which SES was indirectly associated with adolescent depressive symptoms.

As hypothesized, we found that SES was associated with adolescent depressive symptoms through maternal care and adolescent SOC sequentially. This supported the existing theory and empirical findings that the external context including distal (such as SES) and proximal factors (such as maternal care) can contribute to the development of internal resources (such as adolescent SOC) (Benson et al., 2006) and then individual psychosocial development (such as adolescent depressive symptoms) (Benson, 2003; Benson et al., 2006). It should be noted that, because of the relatively small direct effect (β = –0.11), the effects of maternal care and adolescent SOC on the relationships between family SES and adolescent depressive symptoms need to be further examined in future research.

Of note, gender and school stage were not found to moderate the indirect pathways of maternal care and adolescent SOC on the relationship between SES and adolescent depressive symptoms. This may suggest that although there were gender and school stage differences in main variables, the relational mechanism between SES, maternal care, SOC and depressive symptoms was similar for both boys and girls in middle schools and in high schools. Given that few studies have examined school stage as a moderator of the indirect pathways from external context to individual development outcome, further research is required to reach strong conclusions.

### Limitations and Recommendations for Future Research

One limitation of the present study is that we focused on the relationships of SES and adolescents’ perceived depressive symptoms, maternal care/control and SOC, therefore adolescent self–report was used to obtain all study variables. It was possible that the results were limited by relying solely on a single reporter. Using multiple informant measures may reduce the influence of common method variance in future research. This was a community sample and we used a depressive symptoms measure rather than diagnostic assessments of depression, therefore any conclusions cannot be generalized to a clinical population. Further research could employ clinical populations to examine these relationships further. The second limitation is that we used cross–sectional data to examine the indirect relationships, meaning interpretation of the indirect effects of maternal care and adolescent SOC on the relationship between SES and adolescent depressive symptoms should be cautious and no causal conclusions of relationships can be established. Longitudinal designs should be used to establish the sequential nature of the relationship between SES, parenting, SOC and depressive symptoms in future work. Thirdly, our findings were based on the data reported by participants mostly in low– and middle– income families, which could limit the ability to generalize to participants from higher income families. Finally, it is generally known that mothers tend to have more emotional interaction with their children compared to fathers in both eastern and western societies. Especially in Chinese cultures, due to the historical tradition and social division of labor, of “men working outside while women taking care of the family inside,” it is reasonable to assume that mothers in China are involved much more in their child’s life relative to fathers. Furthermore, Liu and Wang (2015) also found that maternal parenting behaviors rather than paternal parenting influenced children’s internalizing problems. Therefore the present study only focused on the effect of maternal care and control. It should be noted that both mothers and fathers play their unique role in children’s development (Milevsky et al., 2007). In the past two decades, Chinese economic development has led to an increase in the number of women who work, with fathers gradually becoming more engaged in their children’s daily lives (Zhang, 1999). Indeed, including both maternal and paternal parenting in the same model may illustrate a more complete picture of how parental behavior is related to adolescent development. Further studies should endeavor to include maternal as well as paternal parenting.

Despite these limitations, this current study broadened earlier research by investigating whether SES was associated with adolescent depressive symptoms through maternal care and adolescent SOC separately and sequentially on a Chinese sample. The present study had both theoretical and practical implications. Although family SES has long been implicated as an important determinant of adolescent depression (Bøe et al., 2014), investigations exploring relational mechanisms between SES, parenting, SOC and adolescent depressive symptoms, particularly in the Chinese culture, were relatively scarce. In particular, we found that SES was associated with adolescent depressive symptoms indirectly not only through maternal care separately but also through maternal care and adolescent SOC sequentially. Findings highlight that more attention should be given to low– and middle– income families where children are more likely to experience negative maternal parenting and may be more likely to experience depressive symptoms.

## Ethics Statement

This study was carried out in accordance with the recommendations of the Institutional Review Board of Shandong Normal University (Jinan, China). The protocol was approved by the Institutional Review Board of Shandong Normal University. The parents of all participants signed written informed consent in accordance with the Declaration of Helsinki and its later amendments.

## Author Contributions

FX wrote and revised the whole manuscript. WC wrote and revised the whole manuscript. MP wrote some sections and gave suggestions and revised and polished the whole manuscript. TX collected data of this manuscript and consulted relevant literature.

## Conflict of Interest Statement

The authors declare that the research was conducted in the absence of any commercial or financial relationships that could be construed as a potential conflict of interest.
